# The chain-mediating effect of Crp, BMI on the relationship between dietary intake of live microbes and hyperlipidaemia

**DOI:** 10.1186/s12944-024-02107-y

**Published:** 2024-05-03

**Authors:** Jingyi Chen, Shuhua Fang, Jinlin Huo, Nian Yang

**Affiliations:** 1 Department of Pharmacy, Nanjing Lishui People’s Hospital, Zhongda Hospital Lishui Branch Southeast University, Nanjing, 211200 China; 2 Institute of Precision Medicine, The First Affiliated Hospital of Shantou University Medical College, Shantou, Guangdong 515041 China

**Keywords:** Live microbes, Hyperlipidaemia, Obesity, Inflammation, NHANES

## Abstract

**Background:**

Inflammation and obesity are the risk factors for hyperlipidaemia. Nonetheless, research regarding the association between dietary live microbes intake and hyperlipidaemia is lacking. Therefore, this study focused on revealing the relationship between them and mediating roles of inflammation and obesity.

**Methods:**

Totally 16,677 subjects were enrolled from the National Health and Nutrition Examination Survey (NHANES) (1999–2010 and 2015–2020). To explore the correlation between live microbes and hyperlipidaemia as well as blood lipid levels, respectively, multiple logistic regression and linear regression were employed. Furthermore, the mediating roles of body mass index (BMI), C-reactive protein (Crp) and their chain effect were explored through mediating analysis.

**Results:**

High dietary live microbes intake was the protective factor for hyperlipidaemia. In addition, high dietary live microbes intake exhibited a positive relationship to the high-density lipoprotein cholesterol (HDL-C) among males (β = 2.52, 95% CI: 1.29, 3.76, *P* < 0.0001) and females (β = 2.22, 95% CI: 1.05, 3.38, *P* < 0.001), but exhibited a negative correlation with triglyceride (TG) levels in males (β = -7.37, 95% CI: -13.16, -1.59, *P* = 0.02) and low-density lipoprotein cholesterol (LDL-C) levels in females (β = -2.75, 95% CI: -5.28, -0.21, *P* = 0.02). Crp, BMI and their chain effect mediated the relationship between live microbes with HDL-C levels. Moreover, BMI and the chain effect mediated the relationship between live microbes with LDL-C levels.

**Conclusion:**

Dietary live microbes intake is related to a lower hyperlipidaemia risk. Crp, BMI and their chain effect make a mediating impact on the relationship.

**Supplementary Information:**

The online version contains supplementary material available at 10.1186/s12944-024-02107-y.

## Introduction

Cardiovascular disease (CVD) is a primary factor resulting in death among the US adults. The risk of CVD increases by approximately twice in patients with hyperlipidaemia in relative to those with normal cholesterol levels [1]. Hyperlipidaemia is becoming a more common issue in the Europe, the USA, and the developing countries. Hyperlipidaemia is a disease characterized by disruption in lipid metabolism, leading to the irregular levels of lipids, including the decreased high-density lipoprotein cholesterol (HDL-C), increased low-density lipoprotein cholesterol (LDL-C), total cholesterol (TC) and triglycerides (TG) levels [2]. Early intervention of hyperlipidaemia is crucial for mitigating the risk of CVD and preventing premature death. Statins are the primary lipid-lowering drugs. However, limitations of statins, including treatment resistance, intolerable adverse events, and insufficient adherence, have led to poor treatment outcomes [1]. Therefore, a substantial proportion of patients need adjuvant therapy to control hyperlipidaemia.

Some research suggests that dietary intake of live microbes is beneficial for human health [3]. Live microbes from dietary intake can promote intestinal activity and decrease disease susceptibility through integration with the resident gut microbiota [4]. Fermented foods containing live microbes, including dairy products, can improve hyperlipidaemia [5, 6]. Moreover, live microbes can be found in a diverse range of foods like unpeeled fruits, vegetables and meats [7]. However, the association between hyperlipidaemia and dietary live microbes intake has not been clearly explored yet.

Obesity has become a global epidemic. According to a lot of epidemiological studies, obesity is a causative factor for many non-communicable diseases, including diabetes mellitus (DM), hyperlipidaemia and other CVDs [8, 9]. Chronic low-grade inflammation can be usually detected in metabolic diseases and obesity [10]. Inflammation may represent a biological mechanism underlying obesity-related diseases. In fact, many inflammatory markers are related to a higher risk of adverse outcomes among people who have obesity-related diseases [11]. Based on a meta-analysis, body mass index (BMI) is positively related to C-reactive protein (Crp), the systemic inflammation marker [12]. In addition, it has been reported that dietary live microbes intake can mitigate inflammation and obesity [13–15]. Therefore, it may reduce the incidence of hyperlipidaemia through alleviating inflammation or obesity.

In this study, it is hypothesized that high dietary live microbes intake shows a relationship to a reduced risk of hyperlipidaemia, and that Crp, BMI and their chain effect may exert mediating roles. To test this hypothesis, the correlation between dietary live microbes intake and hyperlipidaemia was explored, and whether and to what extent the relationship between blood lipid levels and dietary live microbes intake is mediated by BMI or Crp was explored in accordance with the National Health and Nutrition Examination Survey (NHANES) (1999–2010 and 2015–2020), a large-scale cross-sectional study of the USA.

## Methods

### Study population

**Fig. 1:**
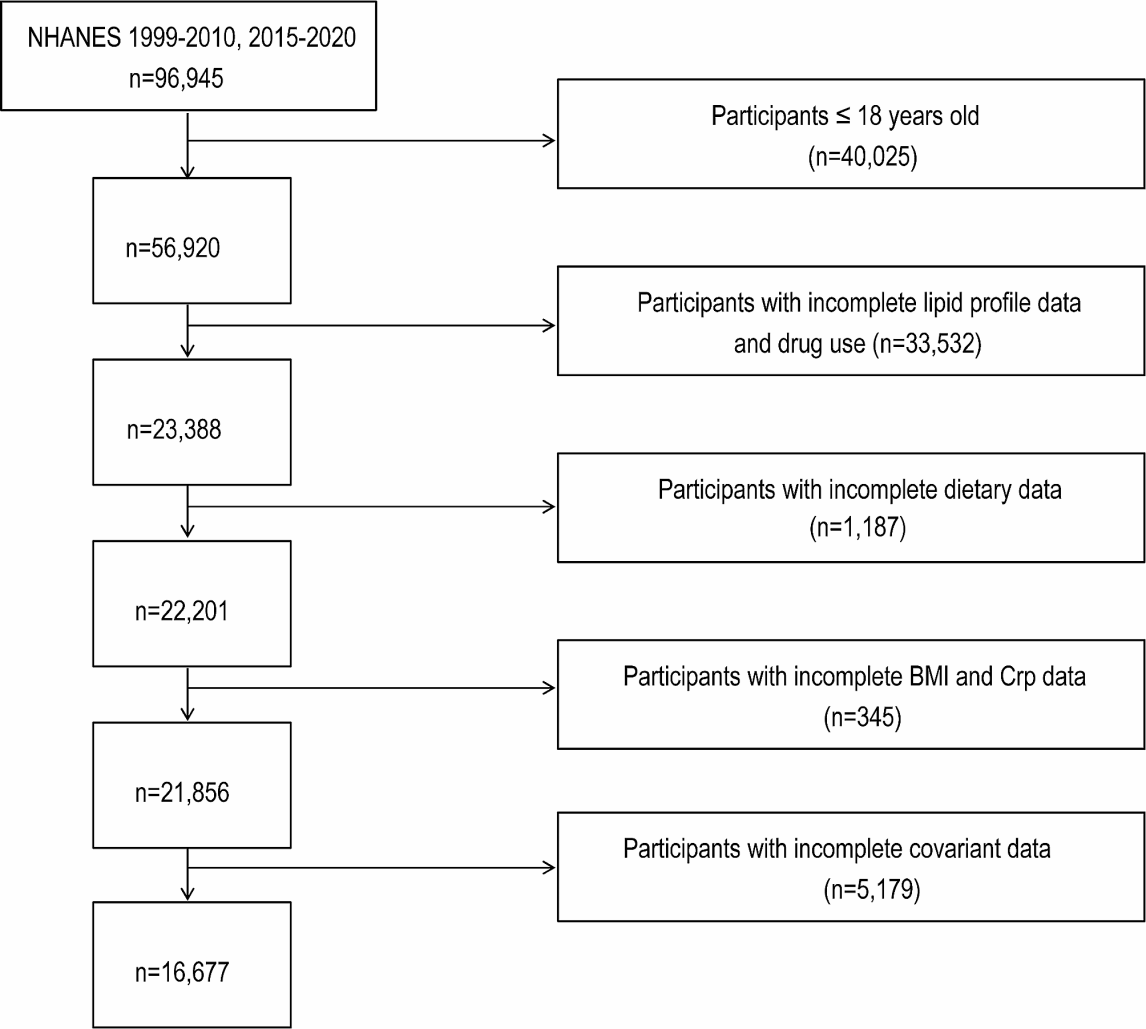
The flow diagram of the participant screening procedure

Data in the present work were acquired in the NHANES (1999–2010 and 2015–2020) (*n* = 96,945). The analysis was restricted to subjects aged 18 years and older (*n* = 56,920). In addition to dietary data (*n* = 23,388), blood lipid levels (HDL-C, LDL-C, TC, TG) and drug use data (*n* = 22,201), BMI and Crp data were also included to estimate the potential mediating effect (*n* = 21,856). Furthermore, other information, which included smoking habits, alcohol consumption, history of hypertension, diabetes, CVD, and stroke, was collected as covariates. Participants missing any one of these variables were eliminated out of analysis. Finally, 16,677 participants were included this study. Figure 1 presents the entire participant screening procedure.

### Assessment of live microbes concentration

In NHANES, dietary intake information was recoded via 24-hour dietary recall interviews. A comprehensive classification system, provided by Sandersm, can estimate quantities of live microbes for food and we analysed 9,388 food codes in the NHANES database [6]. Based on the concentration of live microbes, foods with > 10^7^ CFU/g are classified as high concentra tion, such as unpasteurized fermented foods and probiotic supplements. Those with 10^4–^10^7^ CFU/g are medium concentration, including fresh fruits and vegetables that have not been peeled. In addition, those with < 10^4^ CFU/g are low concentration, referring to pasteurized foods. Participants who only consumed foods with low levels of live microbes were defined as the low dietary live microbe intake group (Low), participants who consumed foods with medium levels of live microbes but not high levels were defined as the medium dietary live microbe intake group (Medium), and participants who consumed foods with high levels of live microbes were defined as the high dietary live microbe intake group (High).

### Hyperlipidaemia assessment

Hyperlipidaemia was classified following the National Cholesterol Education Program Adult Treatment Panel III (NCEP-ATP3). The classification criteria include TC ≥ 200 mg/dL, TG ≥ 150 mg/dL, HDL-C < 40 and < 50 mg/dL in males and females, or LDL-C ≥ 130 mg/dL. Additionally, individuals who reported taking cholesterol-lowering drugs were also considered to have hyperlipidaemia [16].

### Mediators and covariates

Crp was measured with a blood sample, and it was one of the laboratory tests conducted at the Mobile Examination Center (MEC). Crp is a protein produced in the body when inflammation occurs, and it is measured to assess the level of inflammation. In this study, BMI (< 25.0, [25.0, 30.0], ≥ 30.0) was determined through the division of body weight (in kilograms) by square of height (in meters).

Potential covariates included age (years), sex (male/female), ethnicity (Mexican-American/non-Hispanic white/non-Hispanic black/others), education level (lower than high school/high school/college or higher), family poverty income ratio (PIR) (< 1.3, [1.3–3.5], and > 3.5), smoking status (current/ever/never), and drinking status (current/ever/never). Furthermore, medical history, including hypertension, CVD, DM or prediabetes (pre_DM), and stroke, was included in the analysis. Since cholesterol-lowering drugs make an affect on blood lipids, drug use (none, cholesterol-lowering drugs, and other drugs) was also considered as a covariate.

### Statistical analysis

The NHANES provided the four-year sample weights (wtmec4 year) for 1999–2002 and the four-year sample weights (wtmec2 year) for 2003–2020. The weights were calculated by the following formula: 2/9∗wtmec4 year for 1999–2002 + 7/9 ∗wtmec2 year for 2003–2010, 2015–2020. Continuous data were indicated by means and standard errors, whereas categorical data were presented by frequency and percentages. Participants were classified as three groups based on the dietary live microbes intake levels, namely, low, medium, and high groups.

Three multivariate logistic regression models were adopted to explore the relationship between dietary live microbes intake and hyperlipidaemia. No covariates were included in Model 1. In Model 2, age, gender, ethnicity, educational level, PIR, alcohol drinking, and smoking were adjusted. While in Model 3, variables in Model 2, as well as disease history (diabetes, hypertension, CVDs and stroke) and drug use were adjusted. In addition, relationship between dietary live microbes intake groups and blood lipid levels (HDL-C, LDL-C, TG, and TC) was also analyzed through multivariate linear regression. The above analyses were performed using R (version 4.3.1). To explore whether and to what extent the association between blood lipid levels and dietary live microbes intake is mediated by BMI or Crp, the mediation models (bootstrap test with 2000 iterations) were also conducted. In the mediation models, low, medium, and high live microbes intake groups were coded as 0, 1, and 2, respectively, to represent different categorical levels for analysis. Mediation analyses were performed using Stata (version 16.0.2). *P* < 0.05 indicated significant difference.

## Results

### Basic characteristics

Table 1 displays basic characteristics of 16,677 participants, representing 60,188,692 individuals in the USA. Participants were grouped according to the dietary live microbes intake level. Compared to the low group, subjects of the high group were older, women, non-Hispanic white, with higher education degrees, more income, normal weight, non-smokers, current drinkers, without hypertension, stroke, CVD, DM or hyperlipidaemia, with elevated HDL-C, and reduced TC, TG and LDL-C levels (all *P* < 0.05).

**Table 1:** The baseline characteristics of participants by dietary intake of live microbes groups

Variables	Low	Medium	High	P
	5675(31.52)	7426(42.85)	3576(25.63)	
**Age**	44.69(0.28) ^a^	48.02(0.35) ^b^	46.71(0.45) ^c^	< 0.0001
**Gender**				< 0.0001
Female	2681(46.28)	3868(52.40)	2037(56.35)	
Male	2994(53.72)	3558(47.60)	1539(43.65)	
**Ethnicity**				< 0.0001
white	2325(65.22)	3446(70.20)	1995(78.89)	
black	1575(15.31)	1271( 9.15)	444( 5.48)	
mexican	871(7.14)	1611(9.58)	555(5.88)	
other	904(12.34)	1098(11.07)	582( 9.75)	
**Educational level**				< 0.0001
Less than high school	1672(20.55)	1865(15.04)	594( 9.76)	
High school	1452(28.20)	1692(24.38)	756(21.69)	
College or higher	2551(51.24)	3869(60.58)	2226(68.55)	
**BMI**				< 0.001
<25	1619(30.31)	2147(31.48)	1109(32.97)	
25-29.9	1832(31.35)	2599(33.60)	1233(35.10)	
>=30	2224(38.34)	2680(34.92)	1234(31.93)	
**PIR**				< 0.0001
<1.3	1902(24.06)	1889(16.64)	752(13.79)	
1.3–3.5	2328(39.06)	3009(36.86)	1269(31.89)	
>3.5	1445(36.89)	2528(46.49)	1555(54.32)	
**Smoking**				< 0.0001
Never	2831(48.44)	4074(53.92)	2021(56.19)	
Former	1297(22.71)	2049(27.96)	992(28.28)	
Current	1547(28.85)	1303(18.12)	563(15.54)	
**Drinking**				< 0.0001
Never	797(11.28)	985(10.77)	400( 8.14)	
Former	968(14.89)	1121(12.62)	441(11.04)	
Current	3910(73.84)	5320(76.61)	2735(80.82)	
**Hypertension**				0.02
No	3281(63.65)	4366(63.43)	2280(67.19)	
Yes	2394(36.35)	3060(36.57)	1296(32.81)	
**Stroke**				0.002
No	5411(96.83)	7201(97.77)	3476(97.94)	
Yes	264(3.17)	225(2.23)	100(2.06)	
**CVD**				0.002
No	5013(91.11)	6652(91.89)	3263(93.19)	
Yes	662(8.89)	774(8.11)	313(6.81)	
**DM**				0.03
No	2502(50.06)	3281(49.60)	1699(50.69)	
pre_DM	2159(36.57)	2797(36.28)	1315(38.22)	
DM	1014(13.37)	1348(14.12)	562(11.09)	
**Hyperlipidaemia**				0.003
No	1540(28.07)	1903(27.21)	1066(31.61)	
Yes	4135(71.93)	5523(72.79)	2510(68.39)	
**Drug Use**				< 0.001
None	2531(46.24)	3111(41.11)	1552(42.22)	
Others	2213(39.38)	2961(43.06)	1399(40.74)	
Yes	931(14.38)	1354(15.82)	625(17.04)	
**TC (mg/dL)**	193.18(0.83) ^a^	195.62(0.68) ^b^	194.76(0.84) ^b^	0.04
**TG (mg/dL)**	122.72(1.16) ^a^	123.01(1.18) ^a^	115.28(1.49) ^b^	< 0.001
**LDL-C (mg/dL)**	116.86(0.69)	116.34(0.57)	115.13(0.77)	0.27
**HDL-C (mg/dL)**	51.79(0.33) ^a^	54.68(0.29) ^b^	56.57(0.36) ^c^	< 0.0001

BMI: Body Mass Index; CVD: Cardiovascular disease; DM: diabetes mellitus; HDL-C: high-density lipoprotein cholesterol; LDL-C: low-density lipoprotein cholesterol; RIP: ratio of family income to poverty; TC: total cholesterol; TG: triglyceride. For continuous variables, ANOVA was used for means comparison, followed by post-hoc analyses for pairwise comparisons. Different letters indicate significant differences between groups. For categorical variables, contingency tables and chi-squared tests were conducted

### Relationship between dietary live microbes intake and hyperlipidaemia

**Fig. 2:**
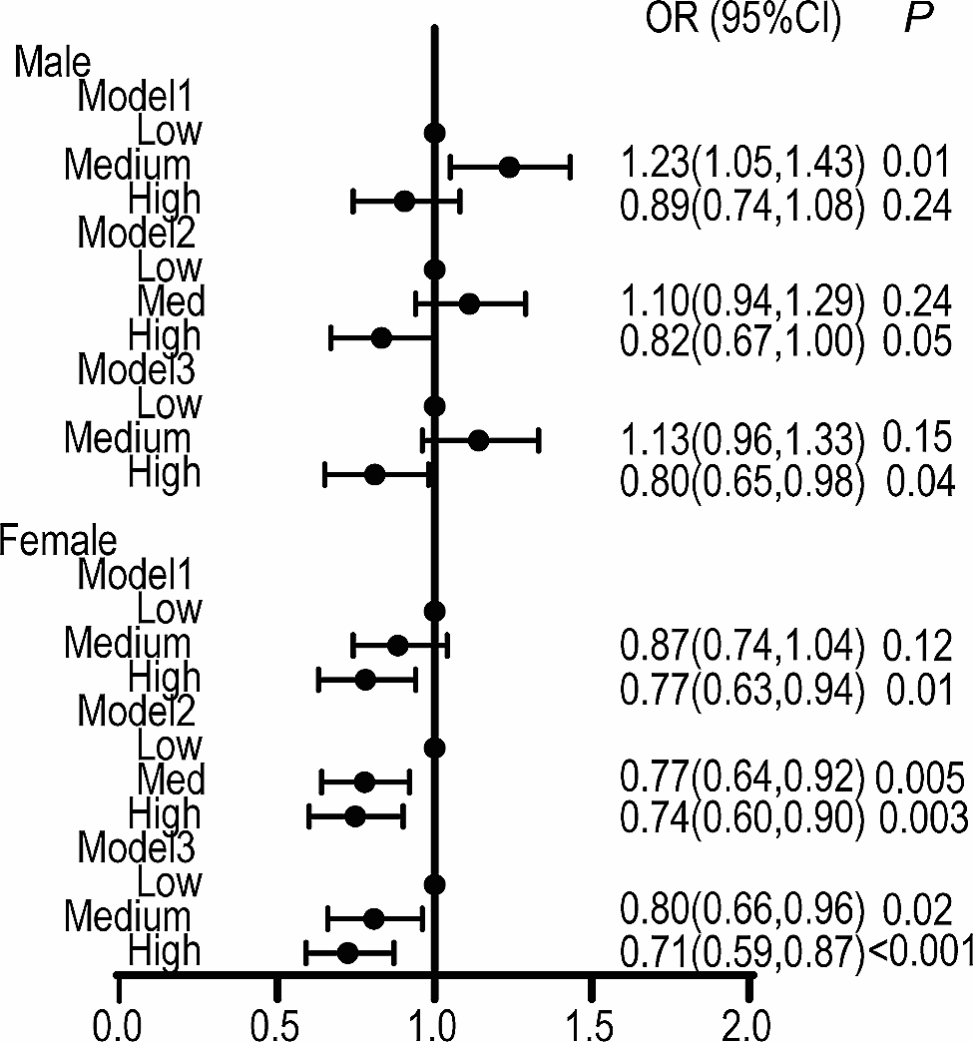
Association between dietary intake of live microbes and hyperlipidaemia. Model 1 included no covariates. Model 2 adjusted for age, gender, ethnicity, educational level, PIR, alcohol drinking status and smoking status. Model 3 was adjusted for the Model 2 variables as well as diabetes, hypertension, CVD, stroke and drug use

Figure 2 illustrates the correlation between dietary live microbes intake with hyperlipidaemia. In females, the odds ratio (OR) (95% CI) for hyperlipidaemia significantly declined from the medium group to the high group in all the three models. It suggests that medium and high dietary live microbes intake levels were protective factors for hyperlipidaemia in females (OR (95% CI) _(Medium)_ = 0.80 (0.66, 0.96); OR (95% CI) _(High)_ = 0.71 (0.59, 0.87)). In males, high dietary liver microbes intake was the protective factor for hyperlipidaemia, with OR (95% CI) of 0.80 (0.65, 0.98) in Model 3.

**Fig. 3:**
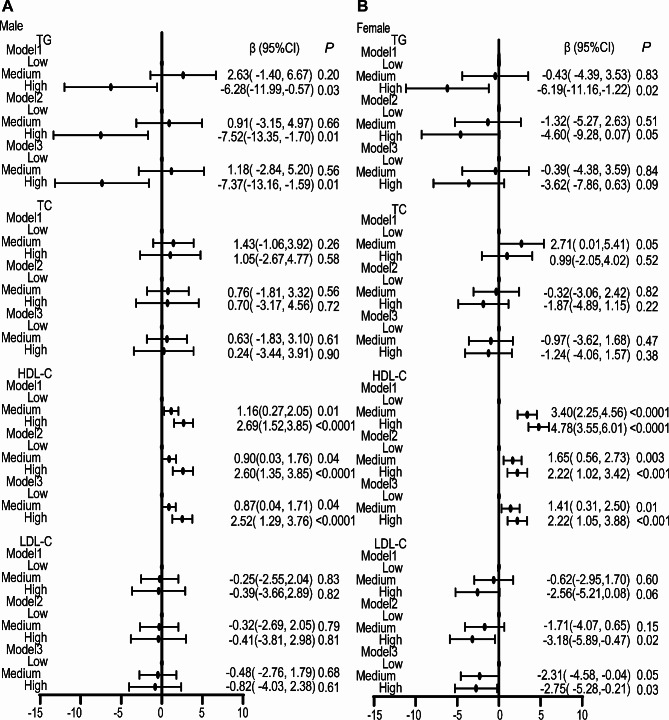
Association between dietary intake of live microbes and serum lipid profiles in males (A) and females (B). Model 1 included no covariates. Model 2 adjusted for age, gender, ethnicity, educational level, PIR, alcohol drinking status, smoking status. Model 3 was adjusted for the Model 2 variables as well as diabetes, hypertension, CVD, stroke and drug use

Moreover, multivariable linear regression models were adopted for evaluating relationship between dietary live microbes intake and blood lipid levels (HDL-C, LDL-C, TC and TG) (Fig. 3). As shown in multivariable linear regression models, after the adjustment for all covariates, the high group still showed significantly positive association with HDL-C levels among males (β = 2.52, 95% CI: 1.29, 3.76, *P <* 0.0001) and females (β = 2.22, 95% CI: 1.05, 3.88, *P* < 0.001). For males, the high group was significantly negatively correlated with TG levels after the adjustment for all covariates (β = -7.37, 95% CI: -13.16, -1.59, *P* = 0.01). In females, the high group was significantly negatively related to LDL-C levels after all covariates were adjusted (β = -2.75, 95% CI: -5.28, -0.21, *P* = 0.03).

### The mediating roles of Crp and BMI in the correlation between dietary live microbes intake and HDL-C or LDL-C

**Fig. 4:**
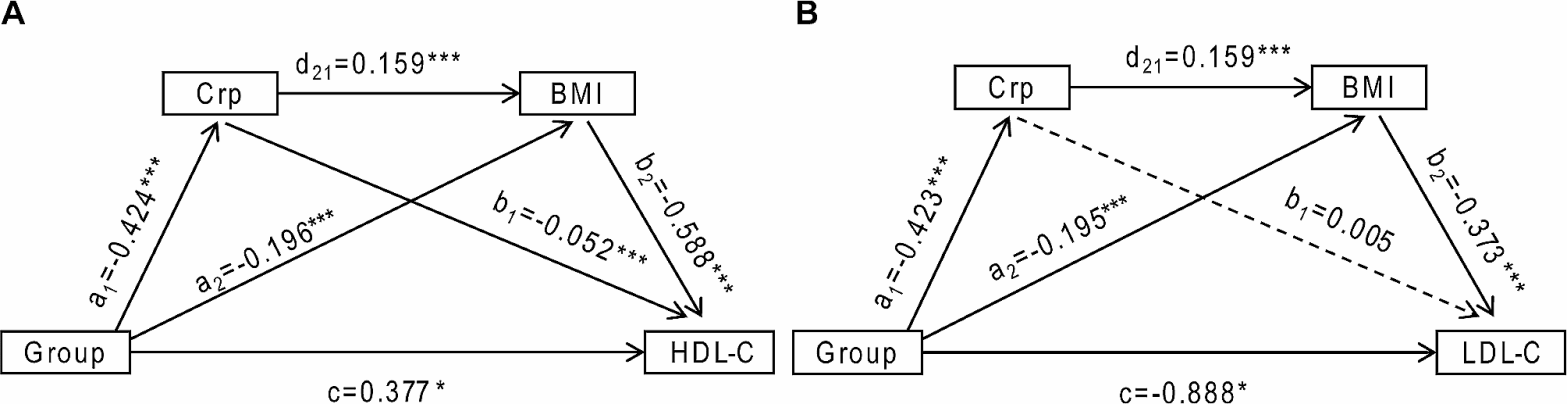
Association of dietary intake of live microbes (X) and HDL-C or LDL-C (Y) mediated by Crp (M1) and BMI (M2). a1 represents the effect of M1 on X; a2 represents the effect of M2 on X; b1 represents the effect of Y on M1; b2 represents the effect of Y on M2; d21 represents the effect of M2 on M1; c represents the direct effect. Solid lines indicate statistically significant associations; dashed lines indicate no statistically significant associations. Adjusted for age, gender, ethnicity, educational level, PIR, alcohol drinking status, smoking status, as well as diabetes, hypertension, CVD stroke and drug use. (A) HDL-C; (B) LDL-C

According to the results mentioned above, mediation analyses were carried out to explore the roles of Crp and BMI, separately and jointly, in the association between dietary live microbes intake and HDL-C or LDL-C. Figure 4 and Table 2 present the results. After adjusting for all covariates, the total effect demonstrated that dietary live microbes intake was significantly related to HDL-C or LDL-C (*P* < 0.05). Both Crp and BMI independently mediated the significant relationship between dietary live microbes intake and HDL-C (*P* < 0.05), suggesting that Crp and BMI acted as the independent mediators in the relationship between dietary live microbes intake with HDL-C (β_Crp_ = 0.021, 95% CI: 0.004, 0.040; β_BMI_ = 0.115, 95% CI: 0.047, 0.183), with the respective mediating proportions of 3.97% and 20.80%. In addition, the chain effect of Crp and BMI on the correlation between dietary live microbes intake and HDL-C levels was also analyzed. As a result, the mediating effect was of statistical significance (β_joint_ = 0.040, 95% CI: 0.021, 0.058), with the mediating proportion of 7.15%. Furthermore, the effect of Crp and BMI on the relationship between dietary live microbes intake and LDL-C levels was also examined, which showed that BMI independently mediated the relationship between dietary live microbes intake and LDL-C levels (β_BMI_ = -0.073, 95% CI: -0.121, -0.024), while Crp did not. However, the effect mediated by Crp and BMI jointly was significant (β_joint_ = -0.025, 95% CI: -0.036, -0.014), with the mediating proportions of BMI and Crp _(BMI and Crp)_ being 7. 40% and 2.53%, respectively.

**Table 2:** The mediating proportions of Crp and BMI on the association between dietary intake of live microbes and HDL-C or LDL-C

Model Pathways	HDL-C-C			LDL-C		
	β (95%CI)		Proportion Mediated (%)	β (95%CI)		Proportion Mediated (%)
**Direct effect**	0.377(0.019, 0.735)***		68.08	-0.888(-1.516,-0.258)**		90.06
**Indirect effect via Crp**	0.021(0.004, 0.040)***		3.97	-0.002(-0.027,0.022)		/
**Indirect effect via BMI**	0.115(0.047, 0.183)***		20.8	-0.073(-0.121,-0.024)***		7.40
**Indirect effect via Crp and BMI**	0.040(0.021, 0.058)***		7.15	-0.025(-0.036,-0.014)***		2.53
**Total effect**	0.554(0.158, 0.949)***		100	-0.986(-1.611,-0.361)***		100

Adjusted for age, gender, ethnicity, educational level, RIP, alcohol drinking status, smoking status, DM, hypertension, CVD, stroke and drug use

** *P* < 0.01; *** *P* < 0.001

## Discussion

Data in this study were obtained from the NHANES (1999–2010 and 2015–2020) for investigating the relationship between dietary live microbes intake and hyperlipidaemia as well as the mediating roles of inflammation (Crp) and obesity (BMI). According to our results, high dietary live microbes intake showed a relationship with a reduced hyperlipidaemia risk. In addition, inflammation, obesity, and their combined effect exhibited partial mediating roles in the relationship between dietary live microbes intake and HDL-C levels. Obesity and the chain effect partially mediated the relationship between dietary live microbes intake and LDL-C levels, but inflammation did not. In the US and European guidelines for hypercholesterolemia management, and hyperlipidaemia is the critical cause of atherosclerotic CVD, and LDL-C accounts for the major target for lipid-lowering treatment [17]. Findings on relationship between dietary live microbes intake and hyperlipidaemia are consistent with previous studies. Meta-analyses supported that probiotics and synbiotics can improve various lipid levels in patients with hyperlipidaemia, DM, and metabolic syndrome [18, 19]. In addition, results in this study suggest that dietary live microbes intake (medium and high groups) protected against hyperlipidaemia in females. Dietary live microbes intake was negatively related to LDL-C levels in females, but there was no significant correlation with LDL-C levels in males. The observed gender discrepancy in lipid metabolism may be due to the metabolism of sex hormones that modulates gut microbiota [20]. Other interventions may be considered for the prevention and treatment of hyperlipidaemia in males.

Crp is a marker of inflammation. The mediating effect of Crp and BMI on the relationship between dietary live microbes intake and blood lipid levels (HDL-C and LDL-C) was analyzed. The results indicated that dietary live microbes intake was related to inflammation and obesity. Dietary live microbes intake was significantly negatively correlated with inflammation. Inflammation showed a negative relationship to HDL-C levels but positively associated with LDL-C levels. Studies have shown that adipose tissue can induce the occurrence of metabolic syndrome by secreting pro-inflammatory factors. Adipose tissue contains immune cells like macrophages, mast cells, T cells, B cells, and dendritic cells. Obese patients exhibit higher levels of pro-inflammatory markers, including Crp, in adipose tissue. In a mouse experiment, mice fed with *Lactobacillus rhamnosus* and *Lactococcus lactis* gained less weights and had lower rates of hepatic steatosis and inflammation [21]. In addition, probiotic consortia have been demonstrated to be effective in controlling weight gain in obese mice and regulating blood lipids, with elevated HDL-C levels and reduced TC and LDL-C levels [22]. Beneficial live microbes may reduce the lipopolysaccharide (LPS) level produced by gut microbiota, thereby alleviating the LPS-induced tissue and systemic inflammation [23].

Collectively, dietary intake of live microbes probably decreases the hyperlipidaemia risk by affecting inflammation and obesity. Therefore, dietary live microbes may help reduce the occurrence of hyperlipidaemia and thereby reduce the risk of metabolic disease.

### Strengths and limitations

There are several advantages in this study. First, it is the first to explore chain effect of BMI and Crp on the correlation between dietary live microbes intake and hyperlipidaemia. Second, NHANES employed complex stratified sampling to ensure national representativeness. Totally 16,677 participants were recruited into this work, collectively representing 60,188,692 individuals. However, certain limitations are required to be noted. First, owing to the cross-sectional study, it was impossible to establish causation or draw definitive cause-and-effect conclusions. Second, even though most covariates were adjusted for, there were still some unmeasured or challenging-to-assess variables that were beyond control. Third, the NHANES dietary data were obtained through interviews, which inevitably introduced some recall bias.

## Conclusion

Dietary live microbes intake is related to a lower hyperlipidaemia risk, and Crp, BMI and their chain effect play mediating roles in this relationship. Therefore, the prevention of hyperlipidaemia through high dietary intake of live microbes provides an economically viable strategy for clinicians to manage patients with hyperlipidaemia.

### Electronic supplementary material

Below is the link to the electronic supplementary material.

Supplementary Material 1

Supplementary Material 2

## Data Availability

No datasets were generated or analysed during the current study.

## Abbreviations

BMI: Body Mass Index
Crp: C-reactive protein
CVD: Cardiovascular disease
DM: Diabetes mellitus
HDL-C: high-density lipoprotein cholesterol
LDL-C: Low-density lipoprotein cholesterol
LPS: Lipopolysaccharide
NCEP-ATP3: National Cholesterol Education Program Adult Treatment Panel III
NHANES: National Health and Nutrition Examination Survey
RIP: Ratio of family income to poverty
TC: Total cholesterol
TG: Triglycerides
