# Supplementary material for: The chain-mediating effect of Crp, BMI on the relationship between dietary intake of live microbes and hyperlipidaemia
Source: Lipids Health Dis. 2024 May 3;23:130. doi: 10.1186/s12944-024-02107-y (PMC11067115; doi:10.1186/s12944-024-02107-y)
Supplement: Supplementary file 1 — Supplementary Material 1 [file 12944_2024_2107_MOESM1_ESM.pdf]

EDITORIAL

This document certifies that the following manuscript was edited for proper English language by one or more of the highly qualified native English speaking editors at Boyi Translation Co.,Ltd. Tangshan, China.

Manuscript title:

The Chain-Mediating Effect of Crp, BMI on the Association between Dietary Intake of Live Microbes and Hyperlipidaemia

Authors

Jingyi Chen , Shuhua Fang , Jinlin Huo , Nian Yang

Date Issued

March 11,2024

唐山博泽翻译有限公司
1302230100543
