# Supplementary material for: The chain-mediating effect of Crp, BMI on the relationship between dietary intake of live microbes and hyperlipidaemia
Source: Lipids Health Dis. 2024 May 3;23:130. doi: 10.1186/s12944-024-02107-y (PMC11067115; doi:10.1186/s12944-024-02107-y)
Supplement: Supplementary file 2 — Supplementary Material 2 [file 12944_2024_2107_MOESM2_ESM.pdf]

5 ok MS_NY.docx

by User User

Submission date: 16-Mar-2024 12:52PM (UTC+0700)
Submission ID: 2321813902
File name: 5_ok_MS_NY.docx (24.06K)
Word count: 2674
Character count: 15692

The Chain-Mediating Effect of Crp, BMI on the Relationship between Dietary Intake of Live Microbes and Hyperlipidaemia

Abstract

Background: Inflammation and obesity are the risk factors for hyperlipidaemia. Nonetheless, studies regarding the association between dietary live microbes intake and hyperlipidaemia is lacking. Therefore, this study focused on revealing the relationship between them and mediating role of inflammation and obesity.

Methods: Totally 16,677 subjects were enrolled from the National Health and Nutrition Examination Survey (NHANES) (1999-2010 and 2015-2020). To explore the correlation between live microbes and hyperlipidaemia as well as blood lipid levels, respectively, multiple logistic regression and linear regression were employed. Furthermore, the mediating role of body mass index (BMI), C-reactive protein (Crp) and their chain effect was explored through mediating analysis.

Results: High dietary live microbes intake was the protective factor for hyperlipidaemia. In addition, high dietary live microbes intake exhibited a positive relationship to the high-density lipoprotein cholesterol (HDL-C) among males (β = 2.52, 95% CI: 1.29, 3.76, $P < 0.0001$) and females (β = 2.22, 95% CI: 1.05, 3.38, $P < 0.001$), but exhibited a negative correlation with triglyceride (TG) levels in males (β = -7.37, 95% CI: -13.16, -1.59, $P = 0.02$) and low-density lipoprotein cholesterol (LDL-C) levels in females (β = -3.32, 95% CI: -5.28, -0.21, $P = 0.02$). Crp, BMI and their chain effect mediated the relationship between live microbes with HDL-C levels. Moreover, BMI and the chain effect mediated the relationship between live microbes with LDL-C levels.

Conclusion: Dietary live microbes intake is related to a lower hyperlipidaemia risk. Crp, BMI and their chain effect make a mediating impact on the relationship.

Introduction

Cardiovascular disease (CVD) refers to a primary factor resulting in death among the US adults. The risk of CVD increases by approximately twice in patients with hyperlipidaemia in relative to those with normal cholesterol levels [1]. Hyperlipidaemia is becoming a more common issue in the Europe, the USA, and the developing countries. Hyperlipidaemia is a disease characterized by disruption in lipid metabolism, resulting in the irregular levels of various lipids, including the decreased high-density lipoprotein cholesterol (HDL-C), low-density lipoprotein cholesterol (LDL-C), total cholesterol (TC), as well as triglycerides (TG) levels [2]. Early intervention of hyperlipidaemia is crucial for mitigating the risk of CVD and preventing premature death. Statins are the primary lipid-lowering drugs. However, limitations of statins, including treatment resistance, intolerable adverse events, and insufficient adherence, have led to poor treatment outcomes [1]. Therefore, a substantial proportion of patients need to receive adjuvant therapy to control hyperlipidaemia.

Some research suggests that dietary intake of live microbes is beneficial for human health [3]. Live microbes from dietary intake can promote intestinal activity and decrease disease susceptibility through integration with the resident gut microbiota [4]. Fermented foods containing live microbes, including dairy products, can improve hyperlipidaemia [5, 6]. Moreover, live microbes can be found in a diverse range of foods like unpeeled fruits,

vegetables and meats [7]. Nonetheless, the association of hyperlipidaemia with dietary live microbes intake has not been clearly explored yet.

Obesity has become a global epidemic. According to a lot of epidemiological studies, obesity is a causative factor for many non-communicable diseases, including diabetes mellitus (DM), hyperlipidaemia and other CVDs [8, 9]. Chronic low-grade inflammation can be usually detected in metabolic diseases and obesity [10]. Inflammation may represent a biological mechanism underlying obesity-related diseases. In fact, many inflammatory markers are related to a higher risk of adverse outcomes among people who develop obesity-related diseases [11]. Based on a meta-analysis, body mass index (BMI) is positively related to C-reactive protein (Crp), the systemic inflammation marker [12]. In addition, it has been reported that dietary live microbes intake can mitigate inflammation and obesity [13-15]. Therefore, it may reduce the hyperlipidaemia incidence through alleviating inflammation or obesity.

In this study, it is hypothesized that high dietary live microbes intake shows a relationship to a reduced risk of hyperlipidaemia, and that Crp, BMI and their chain effect may exert a mediating role. To test this hypothesis, the correlation between dietary live microbes intake and hyperlipidaemia was explored, and whether and to what extent the relationship between blood lipid levels and dietary live microbes intake was mediated by BMI or Crp was explored in accordance with National Health and Nutrition Examination Survey (NHANES) (1999-2010 and 2015-2020), a large-scale cross-sectional study of the USA.

Methods

Study population

Data in the present work were acquired in the NHANES (1999-2010 and 2015-2020) (n = 96,945). The analysis was restricted to subjects aged 18 years and older (n = 56,920). In addition to dietary data (n = 23,388), blood lipid levels (HDL-C, LDL-C, TC, TG) and drug use data (n = 22,201), Crp data were also included to estimate the potential mediating effect (n=21,856). Furthermore, other information, which included BMI, smoking habits, alcohol consumption, history of hypertension, diabetes, CVD, and stroke, was collected as covariates. Participants missing any one of these variables were eliminated out of analysis. Finally, 16,677 participants were recruited into this study. Figure 1 presents the entire participant screening procedure.

Assessment of live microbes concentration

In NHANES, dietary intake information is recoded via 24-hour dietary recall interviews. A comprehensive classification system, provided by Sandersm, can estimate quantities of live microbes for food and we analysed 9,388 food code in NHANES database [6]. Based on the concentration of live microbes, foods with > 107 CFU/g are classed as high concentra tion, such as unpasteurized fermented food and probiotic supplements. Those with 104-107 CFU/g are medium concentration, including fresh fruits and vegetables that have not been peeled. In addition, those with <104 CFU/g are low concentration, referring to pasteurized foods. Participants who only consumed foods with low levels of live microbes were defined as the low dietary live microbe intake group (Low), participants who consumed foods with medium levels of live microbes but not high levels were defined as the medium dietary live microbe intake group (Medium), and participants who consumed foods with high levels of

live microbes were defined as the high dietary live microbe intake group (High).

Hyperlipidaemia assessment

Hyperlipidaemia was classified following National Cholesterol Education Program Adult Treatment Panel III (NCEP-ATP3). The classification criteria include TC ⩾ 200 mg/dL, TG ⩾ 150 mg/dL, HDL-C ⩽ 40 and < 50 mg/dL in males and females, or LDL-C ⩾ 130 mg/dL. Additionally, individuals who reported taking cholesterol-lowering drugs were also considered as hyperlipidaemia [16].

Mediators and Covariates

Crp was measured with a blood sample, and it was one of the laboratory tests conducted at the Mobile Examination Center (MEC). Crp is a protein produced in the body when inflammation occurs, and is measured to assess the level of inflammation. In this study, BMI (< 25.0, [25.0, 30.0], ⩾ 30.0) was determined through division of body weight (in kilograms) by square of height (in meters).

Potential covariates included age (years), sex (male/female), ethnicity (Mexican-American/non-Hispanic white/non-Hispanic black/others), education degree (lower than high school/high school/college or higher), family poverty income ratio (PIR) (< 1.3, [1.3-3.5], and > 3.5), smoking status (current/ever/never), and drinking status (current/ever/never). Furthermore, medical history, including hypertension, CVD, DM or prediabetes (pre_DM), and stroke, was included in the analysis. Since cholesterol-lowering drugs make an impact on blood lipids, drug use (no, cholesterol-lowering drugs, and other drugs) was also considered as a covariate.

Statistical analysis

The NHANES provided the four-year sample weights (wtmec4yr) for 1999-2002 and the four-year sample weights (wtmec2yr) for 2003-2020. The weights were calculated by the following formula: 2/9*wtmec4yr for 1999-2002 +7/9 *wtmec2yr for 2003-2010, 2015-2020. Continuous data were indicated by means and standard errors, whereas categorical ones by frequency and percentages. Participants were classified as three groups following the dietary live microbes intake levels, namely, low, medium, and high groups.

Three multivariate logistic regression models were adopted for examining relationship between dietary live microbes intake and hyperlipidaemia. No covariates were included in Model 1. In Model 2, age, gender, ethnicity, educational degree, PIR, alcohol drinking, and smoking were adjusted. While in Model 3, variables in Model 2, as well as disease history (diabetes, hypertension, CVDs and stroke) and drug use were adjusted. In addition, relationship between dietary live microbes intake groups and blood lipid levels (HDL-C, LDL-C, TG, and TC) was also analyzed through multivariate logistic regression. The above analyses were performed using R (4.3.1). To explore whether and to what extent the association between blood lipid levels and dietary live microbes intake was mediated by BMI or Crp, the mediation models (bootstrap test with 2000 iterations) were also conducted. In the mediation models, low, medium, and high live microbes intake groups were coded as 0, 1, and 2, respectively, to represent different categorical levels for analysis. Mediation analyses were performed using Stata (16.0.2). $P < 0.05$ stood for significant difference.

Results

Basic characteristics

Table 1 displays basic features of 16,677 participants, representing 60,188,692 individuals in the USA. Participants were grouped according to the dietary live microbes intake level. Compared to low group, subjects of high group were older, women, non-Hispanic white, with higher education degrees, more income, normal weight, non-smokers, current drinkers, without hypertension, stroke, CVD, DM or hyperlipidaemia, with elevated HDL-C, and reduced TC, TG and LDL-C contents (all $P < 0.05$).

Relationship between dietary live microbes intake and hyperlipidaemia

Figure 2 illustrates that there is the correlation between dietary live microbes intake with hyperlipidaemia. For males, dietary live microbes intake was not significantly related to hyperlipidaemia in the adjusted models. In females, the odds ratio (OR) (95% CI) for hyperlipidaemia significantly declined from the medium group to the high group in all the three models. It suggested that medium and high dietary live microbes intake levels were protective factors for hyperlipidaemia in females (OR (95% CI) (Medium) = 0.81 (0.67, 0.97); OR (95% CI) (High) = 0.76 (0.63, 0.94)). In males, high dietary liver microbes intake was the protective factor for hyperlipidaemia, with OR (95% CI) of 0.80 (0.65, 0.98) in Model 3.

Moreover, multivariable linear regression models were adopted for evaluating relationship between dietary live microbes intake and blood lipid levels (HDL-C, LDL-C, TC and TG) (Figure 3). As shown in multivariable linear regression models, after the adjustment for all covariates, the high group still showed significantly positive relation to HDL-C levels among males (β = 2.52, 95% CI: 1.29, 3.76, $P < 0.0001$) and females (β = 2.22, 95% CI: 1.05, 3.38, $P < 0.001$). For males, the high group was significantly negatively correlated with TG levels after the adjustment for all covariates (β = -7.37, 95% CI: -13.16, -1.59, $P = 0.02$). In females, the high group was significantly negatively related to LDL-C levels after all covariates were adjusted (β = -3.32, 95% CI: -5.28, -0.21, $P = 0.02$).

The mediating role of Crp and BMI in the correlation between dietary live microbes intake and HDL-C or LDL-C

According to the results mentioned above, mediation analyses were carried out to reveal the role of Crp and BMI, separately and jointly, in the association between dietary live microbes intake and HDL-C or LDL-C. Figure 4 and Table 2 exhibit results. After adjusting for all covariates, the total effect demonstrated that dietary live microbes intake was significantly related to HDL-C or LDL-C ($P < 0.05$). Both Crp and BMI independently mediated the significant relationship between dietary live microbes intake and HDL-C ($P < 0.05$), suggesting that Crp and BMI acted as the independent mediators in the relationship between dietary live microbes intake and HDL-C (β_{Crp} = 0.021, 95% CI: 0.004, 0.040; β_{BMI} = 0.115, 95% CI: 0.021, 0.058), with the respective mediating proportions of 3.97% and 20.80%. In addition, the chain effect of Crp and BMI on the correlation between dietary live microbes intake and HDL-C levels was also analyzed. As a result, the mediating effect was of statistical significance (β_{joint} = 0.040, 95% CI: 0.021, 0.058), with the mediating proportion of 7.15%. Furthermore, the effect

of Crp and BMI on the relationship between dietary live microbes intake and LDL-C levels was also examined, which showed that BMI independently mediated the relationship between dietary live microbes intake and LDL-C levels, while Crp did not. However, the effect mediated by Crp and BMI jointly was significant (βjoint = -0.025, 95% CI: -0.036, -0.014), with the mediating proportions of BMI and Crp (BMI and Crp) being 7. 40% and 2.53%, respectively.

Discussion

Data in this study were obtained from the NHANES (1999-2010 and 2015-2020) for investigating the relationship between dietary live microbes intake and hyperlipidaemia as well as mediating role of inflammation (Crp) and obesity (BMI). According to our results, high dietary live microbes intake showed a relationship to a reduced hyperlipidaemia risk. In addition, inflammation, obesity, and their combined effect exhibited partial mediating role in the relationship between dietary live microbes intake and HDL-C levels. Obesity and the chain effect partially mediate the relationship between dietary live microbes intake and LDL-C levels, but inflammation does not. In the US and European guidelines for hypercholesterolemia management, hyperlipidaemia is the critical cause of atherosclerotic CVD, and LDL-C accounts for the major target for lipid-lowering treatment [17]. Findings on relationship between dietary live microbes intake with hyperlipidaemia are consistent with previous studies. Meta-analyses support that probiotics and synbiotics can improve various lipid levels in patients with hyperlipidaemia, DM, and metabolic syndrome [18, 19]. In addition, results in this study suggested that dietary live microbes intake (medium and high groups) protected against hyperlipidaemia in females. Dietary live microbes intake was negatively related to LDL-C levels in females, but there was no significant correlation with LDL-C levels in males. The observed gender discrepancy in lipid metabolism can probably be caused by the metabolism of sex hormones that modulates gut microbiota [20]. Other interventions may be considered for the prevention and treatment of hyperlipidaemia in males.

Crp is a marker of inflammation. The mediating effect of Crp and BMI on the relationship between dietary live microbes intake and blood lipid levels (HDL-C and LDL-C) was analyzed. The results indicated that dietary live microbes intake was related to inflammation and obesity. Dietary live microbes intake was significantly negatively correlated with inflammation. While inflammation showed a negative relationship to HDL-C levels but positively associated with LDL-C levels. Studies have shown that adipose tissue can induce the occurrence of metabolic syndrome by secreting pro-inflammatory factors. Adipose tissue contains immune cells like macrophages, mast cells, T cells, B cells, and dendritic cells. Obese patients exhibit higher levels of pro-inflammatory markers, including Crp, in adipose tissue. In a mouse experiment, mice fed with Lactobacillus rhamnosus and Lactococcus lactis gained less weights and had lower rates of hepatic steatosis and inflammation [21]. In addition, probiotic consortia has been demonstrated to be effective in controlling weight gain in obese mice and regulating blood lipids, with elevated HDL-C contents and reduced TC and LDL-C contents [22]. Beneficial live microbes may reduce the lipopolysaccharide (LPS) level produced by gut microbiota, thereby alleviating the LPS-induced tissue and systemic inflammation [23].

Collectively, dietary intake of live microbes probably decreases the hyperlipidaemia risk by affecting inflammation and obesity. Therefore, dietary live microbes may help reduce the occurrence of hyperlipidaemia and thereby reduce the risk of metabolic disease.

Strengths and Limitations

There are several advantages in this study. First, it is the first to explore chain effect of BMI and Crp on the correlation between dietary live microbes intake and hyperlipidaemia. Second, NHANES employed complex stratified sampling to ensure the national representativeness. Totally 16,677 participants were recruited into this work, collectively representing 60,188,692 individuals. However, certain limitations are required to be noted. Firstly, owing to the cross-sectional study, it was impossible to establish causation or draw definitive cause-and-effect conclusions. Secondly, even though most of the covariates were adjusted, there were still some unmeasured or challenging-to-assess variables beyond control. Thirdly, the NHANES dietary data were obtained through interviews, which inevitably introduced some recall errors.

Conclusion

Dietary live microbes intake is related to the lower hyperlipidaemia risk, and Crp, BMI and their chain effect play a mediating role in such relationship. Therefore, the prevention of hyperlipidaemia through high dietary intake of live microbes provides an economically viable strategy for clinicians to manage patients with hyperlipidaemia.

5 ok MS_NY.docx

ORIGINALITY REPORT

10%	3%	4%	2%
SIMILARITY INDEX	INTERNET SOURCES	PUBLICATIONS	STUDENT PAPERS

PRIMARY SOURCES

1	www.ncbi.nlm.nih.gov<br>Internet Source	1%
2	Yiping Liu, Yida Xing, Xiaodan Kong. "Exploratory analysis on the relationship between dietary live microbe intake and arthritis: a national population based cross-sectional study", Frontiers in Nutrition, 2024<br>Publication	1%
3	worldwidescience.org<br>Internet Source	1%
4	docplayer.fr<br>Internet Source	1%
5	bmcpublichealth.biomedcentral.com<br>Internet Source	1%
6	www.mdpi.com<br>Internet Source	1%
7	Baimei He, Qiong Huang, Siqi Li, Wenbin Nan, Qiong Chen, Qiao Yu. "Relationship between dietary live microbe intake and the prevalence of COPD in adults: A Cross-Sectional Study of	1%

NHANES 2013-2018", Research Square Platform LLC, 2024
Publication

8	Li, Michelle Muzhi. "The Association of Postoperative Anemia With Adverse Outcomes in Patients Undergoing Cardiac Surgery: A Retrospective Cohort Study", University of Toronto (Canada), 2023<br>Publication	1%
9	Metabolic Syndrome, 2016.<br>Publication	1%
10	www.researchsquare.com<br>Internet Source	<1%
11	Min Wang, Zhao-hui Huang, Yong-hong Zhu, Shuai Li, Xin Li, He Sun, Ping He, Ya-li Peng, Qiu-Ling Fan. "Association of dietary live microbe intake with diabetic kidney disease in patients with type 2 diabetes mellitus in US adults: a cross-sectional study of NHANES 1999–2018", Acta Diabetologica, 2024<br>Publication	<1%
12	link.springer.com<br>Internet Source	<1%
13	vau.ac.lk<br>Internet Source	<1%
14	www.nature.com<br>Internet Source	<1%

15 X. Wang, J. Li, W. Liang, T. Fan, G. Ruan, Y. Zhang, Z. Zhu, D. Hunter, C. Ding. "SYNOVITIS MEDIATES THE ASSOCIATION BETWEEN BONE MARROW LESIONSAND KNEE PAIN IN OSTEOARTHRITIS:DATA FROM THE FOUNDATION FOR THE NATIONAL INSTITUTE OF HEALTH (FNIH) OSTEOARTHRITIS BIOMARKERS CONSORTIUM", Osteoarthritis and Cartilage, 2022
Publication
<1%

16 journals.plos.org
Internet Source
<1%

17 www.science.gov
Internet Source
<1%

18 Chuanli Yang, Qin Hong, Teng Wu, Yunhe Fan, Xiaobing Shen, Xiushan Dong. "Association between dietary intake of live microbes and chronic constipation in adults", The Journal of Nutrition, 2023
Publication
<1%

19 www.omicsdi.org
Internet Source
<1%

20 Xingwei Huo, Shanshan Jia, Xin Zhang, Lirong Sun, Xueting Liu, Lu Liu, Xianghao Zuo, Xiaoping Chen. "Association of dietary live microbe intake with abdominal aortic calcification in US adults: a cross-sectional
<1%

study of NHANES 2013–2014", Frontiers in Nutrition, 2023
Publication

21	Haoxian Tang, Xuan Zhang, Nan Luo, Jingtao Huang, Yanqiao Zhu. "Association of Dietary Live Microbes and Nondietary Prebiotic/Probiotic Intake With Cognitive Function in Older Adults: Evidence From NHANES", The Journals of Gerontology: Series A, 2023<br>Publication	<1 %

Exclude quotes	Off	Exclude matches	Off
Exclude bibliography	On		
